# Longitudinal effects of antidepressant treatment on resting state functional connectivity in adolescents with major depressive disorder

**DOI:** 10.1192/j.eurpsy.2022.256

**Published:** 2022-09-01

**Authors:** K.H. Lee, J. Shin, J. Lee, J.H. Yoo, J.-W. Kim

**Affiliations:** 1 Seoul National University Hospital, Division Of Child And Adolescent Psychiatry, Department Of Psychiatry, Seoul, Korea, Republic of; 2 Seoul National University Children’s Hospital, Integrative Care Hub, Seoul, Korea, Republic of; 3 Seoul ST. Mary’s Hospital, Department Of Psychiatry, Seoul, Korea, Republic of

**Keywords:** adolescence, major depressive disorder, resting-state functional connectivity, antidepressant treatment

## Abstract

**Introduction:**

Adolescents with major depressive disorder (MDD) often show reduced prefrontal functional connectivity with the subcortical regions than healthy controls (HC) (Tang et al., 2018). However, relatively little is known about longitudinal effects of antidepressant (AD) treatment on resting state functional connectivity (RSFC) in the prefrontal cortex (PFC).

**Objectives:**

This study aimed to investigate abnormal PFC RSFC in MDD adolescents compared to HC and longitudinal effects of AD on PFC RSFC.

**Methods:**

This study included 59 adolescents with MDD and 43 HC. MDD adolescents were treated with escitalopram in an 8 week, open-label trial. The treatment outcome was assessed by Children’s Depression Rating Scale (CDRS-R) and patients showing at least a 40% improvement in CDRS-R scores from baseline to week 8 were defined as “responders”. Functional and T1 images collected before and after treatment were processed using AFNI and Freesurfer. Our seed was the lateral PFC (LPFC, BA46). T-tests and repeated measures ANCOVAs, controlling for age and IQ, were conducted to examine abnormal PFC RSFC and longitudinal effects of AD on LPFC RSFC.

**Results:**

Relative to HC, MDD showed increased LPFC RSFC with the posterior middle temporal gyrus (pMTG) and superior frontal cortex (SFG) involved in attentional networks. Responders showed greater changes in LPFC RSFC with the MTG and SFG after AD treatment compared to non-responders and HC (Figure 1).

**Figure:**
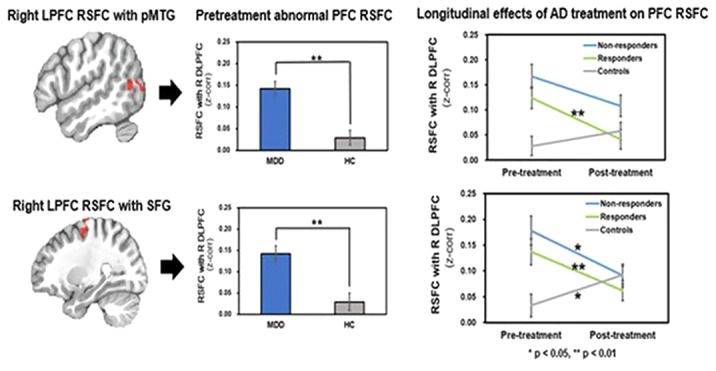

**Conclusions:**

Our finding suggests that reduced LPFC RSFC with the pMTG and SFG reflecting decreased attentional network connectivity may serve as a biomarker to predict AD treatment outcome in adolescents with MDD.

**Disclosure:**

No significant relationships.

